# Xiaotangzhike Pill Attenuates the Progression of Diabetes In Vivo through the Mediation of the Akt/GSK-3*β* Axis

**DOI:** 10.1155/2022/6709506

**Published:** 2022-12-21

**Authors:** Liu Yang, Wei Wang, Yanbo Fan, Zheren Lin, Yisheng Zhang, Qingfeng Zeng

**Affiliations:** Department of Pharmacy, Wuhan Hospital of Traditional Chinese Medicine, Wuhan, Hubei 430050, China

## Abstract

**Background:**

Diabetes seriously threatens the health of people. Traditional Chinese medicine has been proven to inhibit the progression of diabetes. Meanwhile, the Xiaotangzhike pill (XTZK) was known to alleviate the symptom of diabetes. Thus, this research decided to investigate the mechanism underlying the impact of XTZK in diabetes remains unexplored.

**Methods:**

To assess the impact of XTZK in diabetes, in vivo model of diabetes was constructed. The contents of total cholesterol (TC), low-density lipoprotein cholesterol (LDL-C), triglyceride (TG), and high-density lipoprotein cholesterol (HDL-C) in the rats were tested by the commercial kits. In addition, Masson and hematoxylin and eosin (H&E) staining were applied for assessing the histological changes and fibrosis in the rats, respectively. Furthermore, a western blot was applied to assess the protein levels.

**Results:**

Streptozotocin (STZ) significantly increased the levels of area under the curve (AUC), TG, TC, LDL-C, and decreased the contents of HDL-C in rats, while these phenomena were partially reversed by XTZK. In addition, STZ notably induced inflammatory infiltration and fibrosis in the liver tissues of rats, which was greatly restored by XTZK. The levels of aspartate aminotransferase (AST), alanine aminotransferase (ALT), and malondialdehyde (MDA) in the serum of rats were notably upregulated by STZ, while the effect of STZ was markedly abolished by XTZK. Meanwhile, STZ-caused the upregulation of p-Smad2 and *α*-SMA in rats was restored by XTZK. Furthermore, XTZK notably inhibited the progression of Qi and Yin deficiency syndrome in diabetes through the mediation of the Akt/GSK-3*β* axis.

**Conclusion:**

The Xiaotangzhike pill attenuates the progression of diabetes through the mediation of the Akt/GSK-3*β* axis. Hence, our study might supply a novel insight into discovering new strategies against diabetes.

## 1. Introduction

The prevalence of diabetes has been increasing worldwide for over 30 years [1]. In addition, the progression of diabetes could induce stroke, blindness, kidney failure, and lower limb amputation [2]. Diabetes is regarded as a vascular disease, which is verified to be characterized by inflammatory responses due to hypoxia and high glucose (HG) [3]. Diabetic vascular complications contain macrovascular dysfunctions that occur in organs [4, 5]. Over half of patients with diabetes die of some complications that is able to make it a cause of mortality [6, 7]. Nowadays, the major treatments for diabetes are drug administration, while the effects are still not ideal. Hence, it is urgent to discover novel strategies against diabetes.

It has been reported that Chinese herbal compounds exerted antidiabetic effects. For instance, Fufang Fanshiliu decoction could inhibit the progression of type II diabetes mellitus by reducing the insulin resistance [8]; Sun et al. found that Shenlian (SL) decoction could alleviate blood glucose via the mediation of gut microbiota in db/db mice [9]; Zhang et al. indicated that Shenqi compound could inhibit diabetes mellitus through the metabolites and gut microbiota [10]. Meanwhile, the Xiaotangzhike pill is a Chinese herbal compound composed of three medicinal herbs, including Radix Puerariae, Atractylodes Lancea, and Scrophularia ningpoensis [11], and its major components were 3′-hydroxypuerarin, puerarin, 3-methoxy puerarin, and daidzein (Supplementary Figure 1). Moreover, XTZK was able to suppress the progression of type II diabetes mellitus [12]. However, the detailed mechanism by which XTZK inhibits the development of diabetes remains unclear.

Based on the above backgrounds, we investigated the impact of XTZK in diabetes. In addition, this study sought to explore the mechanism underlying the function of XTZK in diabetes. We hope this research would supply new strategies for the treatment of diabetes.

## 2. Materials and Methods

### 2.1. In Vivo Model of Diabetes

Male rats (5–7 weeks old, 200 ± 10 g) were purchased from Chinese Academy of Sciences, Shanghai and placed in the condition of SPF. The ethical committee of Wuhan Hospital of Traditional Chinese Medicine approved this research.

Rats were randomly separated into the control (*n* = 5), STZ (*n* = 5), and STZ + XTZK (drug, *n* = 5) groups. After a high-fat diet (HFD, Biotech HD, Beijing, China) period of 4 weeks, the rats were administered with STZ (25 mg/kg, with 0.05 M citrate buffer) intraperitoneally for constructing the diabetes mellitus model [13]. The rats in the control were fed with a normal diet (Biotech HD). After 48 h of STZ injection, rats in STZ and STZ + XTZK groups were administrated with STZ (25 mg/kg) intraperitoneally, and the control rats were injected intraperitoneally with saline. Three days after STZ injection, the blood sample was obtained from the tail vein to assess glucose levels by using the glucometer (ACCU-CHEK® Active). Rats with glucose levels of 7.8–16.7 mmol/L were diagnosed with diabetes. In addition, rats were fed with HFD for 2 weeks in a row. Meanwhile, rats were orally administrated with XTZK for 4 weeks after the rats were diagnosed with diabetes. After that, the area under the glucose tolerance curve was calculated, and the liver tissues and serum were collected from rats after the rats were sacrificed.

To mimic Qi and Yin deficiency syndrome of diabetes *in vivo*, rats were intraperitoneally injected with STZ (30 mg/kg) once a day for two days, and the same volume of saline were injected into control rats. After the injection of STZ, rats have fasted for 12 h, and then fed with HFD for one week. Subsequently, the blood glucose of rats was measured using a glucometer (ACCU-CHEK® Active). Rats with glucose levels ≥11.1 mmol/L were diagnosed with type II diabetes mellitus. After that, rats in STZ and STZ + drug groups were orally administrated with 13 g/kg multiple drugs (including GreenTangerinePeel, Fructus AurantII and Aconitum carmichaeli) for 6 weeks (once a day). Then, rats in the STZ + drug group were treated with XTZK for 4 weeks. Finally, rats were sacrificed, and the pancreatic tissues and serum were collected for further analysis.

### 2.2. Histopathological Analysis

The paraformaldehyde solution (4%, Solarbio) was applied for tissue fixation. The organs were cut, dehydrated, and embedded to sections (4 *μ*m). Masson trichrome (Solarbio) and H&E were applied for staining the sections. For the purpose of analyzing the injury, images were obtained from a microscope (Nikon, Japan) and determined by Image J (version 1.8.0).

### 2.3. Enzyme-Linked Immunosorbent Assay (ELISA)

The serum of rats was collected for assessing the liver function. The AST and ALT levels in the serum were assessed with commercial kits (Sigma-Aldrich), according to the previous report [14].

### 2.4. Biochemical Parameters Analysis

The contents of TC, blood urea nitrogen (BUN), creatinine, TG, LDL-C, HDL-C, superoxide dismutase (SOD), and MDA in the serum of rats were detected by commercial biochemical detection kits (Sigma-Aldrich) as per the previous report [15].

### 2.5. TUNEL Staining

H_2_O_2_ (2%) was applied to treat liver tissue sections for 10 min, and then sections were exposed to DNase-free proteinase K (20 *μ*g/mL) for 30 min to remove nuclease. Next, the equilibrium buffer (100 *μ*l) was applied for incubating the sections for 10 min, and then sections were exposed to 100 *μ*l TdT enzyme for 60 min at 37°C away from light. Afterwards, the reaction termination was applied to stain the sections for 10 min. DAPI solution (10 mg/mL) was applied to stain the sections for 5 min prior to analysis. The results analyzed by the Image J software (Motic Med 6.0).

### 2.6. Western Blotting

RIPA (Beyotime) was applied for extracting total protein. BCA kit (Beyotime) was applied for protein quantification. SDS-PAGE (10%) was applied for separating the proteins. Afterwards, proteins were transferred onto PVDF membranes (Millipore). Next, primary antibodies targeted against p-Smad2 (1 : 1,000), Smad2 (1 : 1,000), *α*-SMA (1 : 1,000), Bax (1 : 1,000), GSK-3*β* (1 : 1,000), *β*-catenin (1 : 1,000), p-Akt (1 : 1,000), Akt (1 : 1,000), and *β*-actin (1 : 1,000) were applied for membrane incubation overnight at 4°C after blocking. The secondary antibodies (HRP-conjugated, ASPEN; 1 : 5,000) were applied for membrane incubation. ECL (invitrogen) was used for visualizing the protein bands. IPP 6.0 (Image-Pro Plus 6.0) was applied for analyzing the densitometry. All antibodies originated from Abcam.

### 2.7. Immunohistochemistry (IHC) Detection

Paraformaldehyde (4%) was applied for fixing the liver tissues overnight, and then tissues were cut into sections (5 *μ*m-thick) after paraffin-embedded. Paraffin sections were rehydrated after deparaffinized. The sodium citrate buffer was applied to heat the sections for antigen retrieval, and then 3% H_2_O_2_ was applied for section incubation for 25 min. Next, the goat serum was applied for incubating the sections after sections were washed with PBS. Then, primary antibodies (anticollagen II and antifibronectin) were used for incubating the sections overnight. The secondary antibody (HRP-labeled) was used for sample incubation at 37°C for 30 min. Finally, freshly prepared diaminobenzidine (DAB) was used for color development. All antibodies were obtained from Abcam.

### 2.8. Statistical Analysis

Three independent experiments were applied in each group. Moreover, the mean ± standard deviation (SD) was applied for expressing all data. One-way analysis of variance (ANOVA) followed by Tukey's test (multiple groups, Graphpad Prism7) or Student's *t*-test (only 2 groups) was used to compare the differences. *P* < 0.05suggested an obvious change.

## 3. Results

### 3.1. XTZK Significantly Alleviated Dyslipidemia in STZ Rats

**Figure 1:**
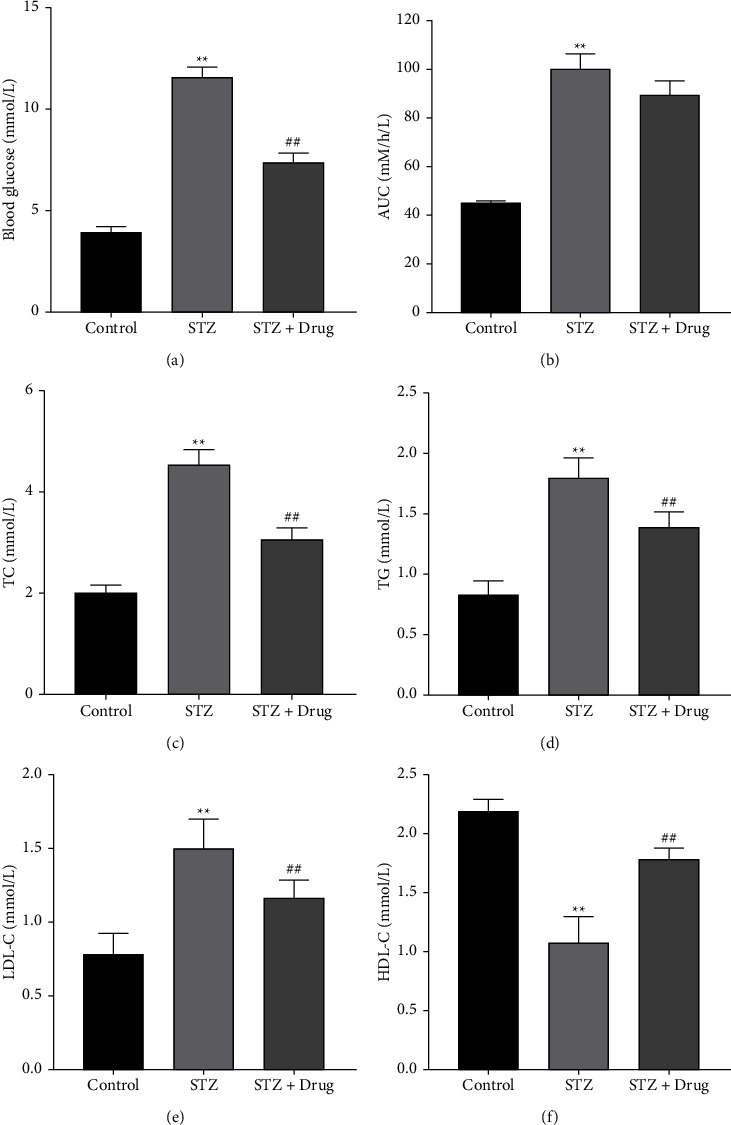
XTZK significantly alleviated dyslipidemia in STZ rats. (a) The level of blood glucose in rats was tested by a glucometer. (b) The area under the glucose tolerance curve of rats was calculated. (c) The concentration of TC in the serum of rats was tested by a biochemical kit. (d) The concentration of TG in the serum of rats was tested by a biochemical kit. (e) The concentration of LDL-C in the serum of rats was tested by a biochemical kit. (f) The concentration of HDL-C in the serum of rats was tested by a biochemical kit. ^*∗∗*^*P* < 0.01 compared to the control. ##*P* < 0.01 compared to STZ. XTZK, Xiaotangzhike pill; STZ, streptozotocin; TC, total cholesterol; TG, triglyceride; LDL-C, low-density lipoprotein cholesterol; HDL-C, high-density lipoprotein cholesterol.

To investigate the function of XTZK in diabetes, rats were injected with STZ, and then administrated with XTZK. As demonstrated in Figures 1(a) and 1(b), the levels of blood glucose and AUC in rats were increased by STZ, which were notably restored in the presence of XTZK. In addition, STZ notably increased the contents of TC, TG, and LDL-C in rats, while this phenomenon was greatly restored by XTZK (Figures 1(c)–1(e)). In contrast, the content of HDL-C in rats was markedly inhibited by STZ, while the inhibitory effect of STZ was rescued by XTZK (Figure 1(f)). Taken together, XTZK significantly restored dyslipidemia in STZ rats.

### 3.2. XTZK Significantly Alleviated Inflammatory Infiltration and Fibrosis in STZ Rats

**Figure 2:**
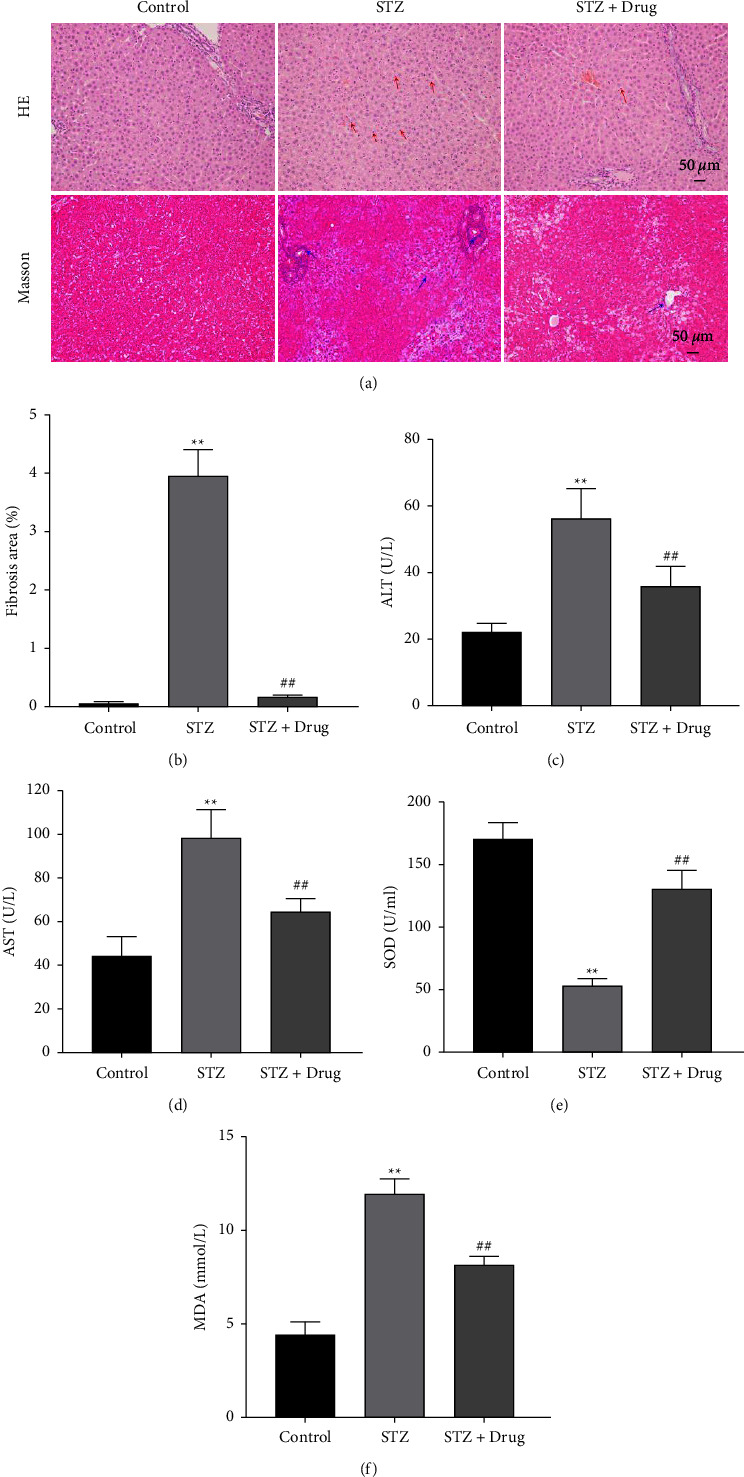
XTZK significantly alleviated inflammatory infiltration and fibrosis in STZ rats. (a), (b) The histological changes in the liver tissues of rats were observed by H&E and Masson staining. (c), (d) The concentrations of ALT and AST in the serum of rats were tested by ELISA. (e), (f) The concentrations of SOD and MDA in the serum of rats were tested by a biochemical kit. ^*∗∗*^*P* < 0.01 compared to the control. ##*P* < 0.01 compared to STZ. XTZK, Xiaotangzhike pill; STZ, streptozotocin; H&E, hematoxylin and eosin; ALT, alanine aminotransferase; AST, aspartate aminotransferase; ELISA, enzyme-linked immunosorbent assay; SOD, superoxide dismutase; MDA, malondialdehyde. Red arrows indicate the inflammatory infiltration, and blue arrows indicate the collagen fiber.

To further investigate the function of XTZK in the liver injury of STZ rats, H&E and Masson staining were performed. As shown in Figures 2(a) and 2(b), STZ significantly induced inflammatory infiltration and fibrosis in rats, while this phenomenon was notably abolished by XTZK. The contents of AST and ALT in rats were notably increased by STZ, which was obviously rescued by XTZK (Figures 2(c) and 2(d)). Meanwhile, STZ notably decreased the level of SOD and increased MDA levels in rats, while this phenomenon was partially restored by XTZK (Figures 2(e) and 2(f)). In summary, XTZK significantly alleviated the inflammatory infiltration and fibrosis in STZ rats.

### 3.3. XTZK Significantly Reversed STZ-Induced Upregulation of Smad2 Signaling in Rats

**Figure 3:**
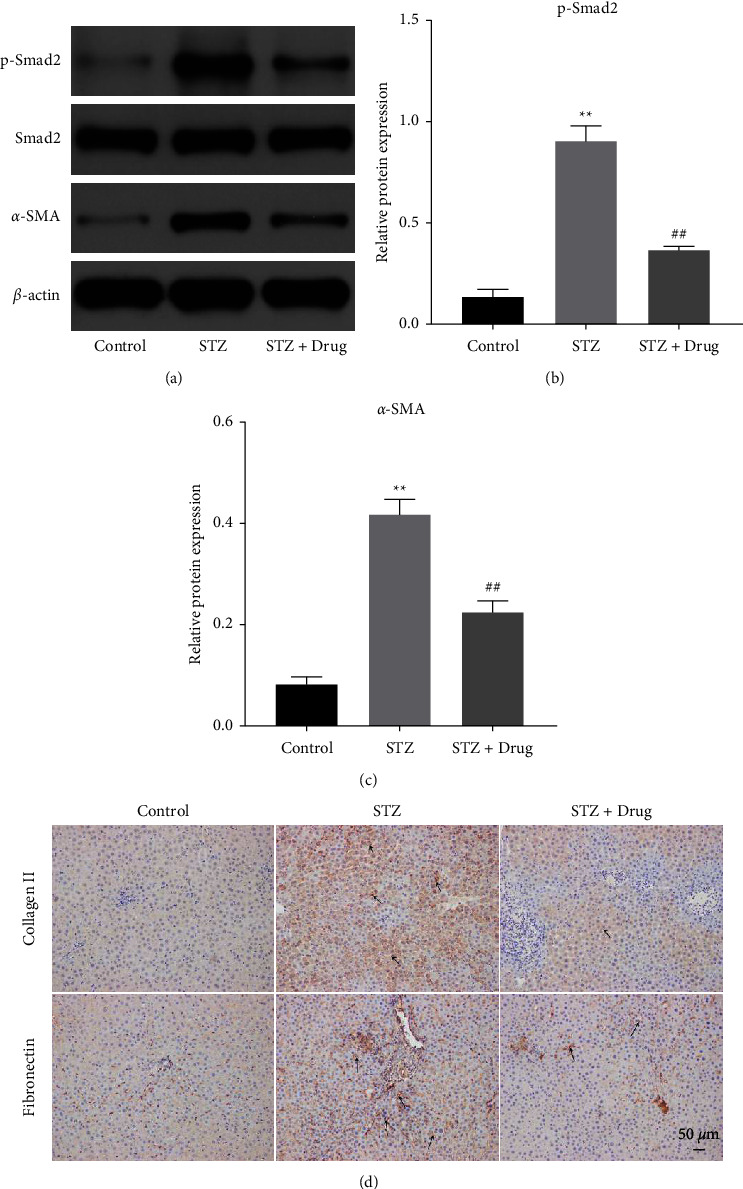
XTZK significantly reversed STZ-induced upregulation of Smad2 signaling in rats. (a) (b), (c) The protein levels of p-Smad2, Smad2, and *α*-SMA in the liver tissues of rats were investigated by a western blot. The relative expressions were quantified normalizing to Smad2 or *β*-actin. (d) The levels of fibronectin and collagen II in the liver tissues of rats were tested by IHC staining. ^*∗∗*^*P* < 0.01 compared to the control. ##*P* < 0.01 compared to STZ. XTZK, Xiaotangzhike pill; STZ, streptozotocin. Black arrows indicate the positive expression of fibronectin and collagen II in the liver tissues of rats.

To investigate the mechanism underlying the function of XTZK in STZ-induced fibrosis, a western blot was used. As demonstrated in Figures 3(a)–3(d), the levels of p-Smad2, *α*-SMA, collagen II, and fibronectin in the liver tissues of rats were significantly upregulated by STZ, while the impact of STZ on these two proteins was significantly inhibited by XTZK. To sum up, XTZK could reverse STZ-induced liver fibrosis in rats through the inactivation of p-Smad2, collagen II, fibronectin, and *α*-SMA.

### 3.4. XTZK Notably Reversed STZ-Induced Kidney Injury in Rats

**Figure 4:**
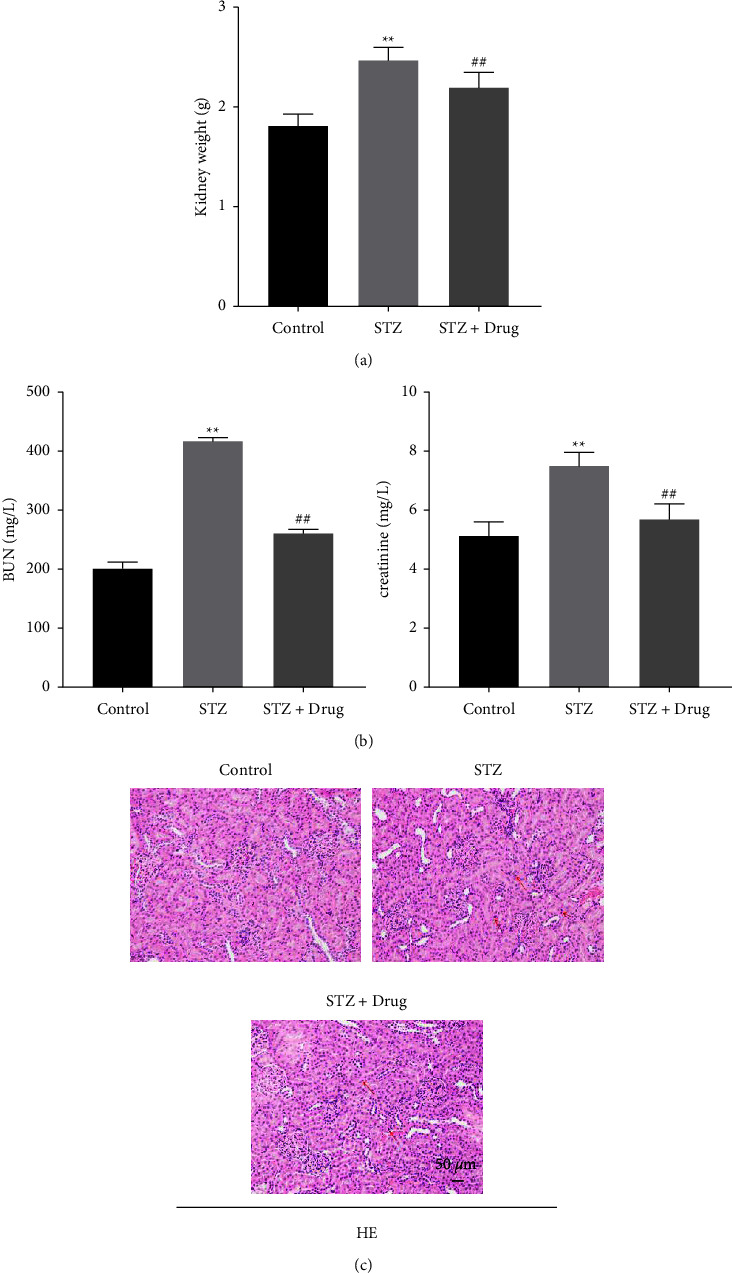
XTZK notably reversed STZ-induced kidney injury in rats. (a) The weight of kidney in rats was recorded. (b) The levels of BUN and creatinine in rats were tested by a biochemical kit. (c) The histological changes in the kidney tissues of rats were observed by H&E staining. ^*∗∗*^*P* < 0.01 compared to the control. ##*P* < 0.01 compared to STZ. XTZK, Xiaotangzhike pill; STZ, streptozotocin; BUN, blood urea nitrogen; H&E, hematoxylin and eosin. Red arrows indicate the inflammatory infiltration.

To investigate the kidney nephrotoxicity in rats, the weight of kidney tissues was tested. As shown in Figure 4(a), STZ significantly increased the weight of kidney tissues in rats, while XTZK reversed this phenomenon. The levels of BUN and creatinine in the serum of rats were notably elevated by STZ, which were declined by XTZK (Figure 4(b)). Furthermore, STZ-induced inflammatory infiltration in the kidney tissues of rats was alleviated by XTZK (Figure 4(c)). In summary, XTZK notably reversed STZ-induced kidney injury in rats.

### 3.5. XTZK Significantly Reversed STZ-Induced Apoptosis in Rats

**Figure 5:**
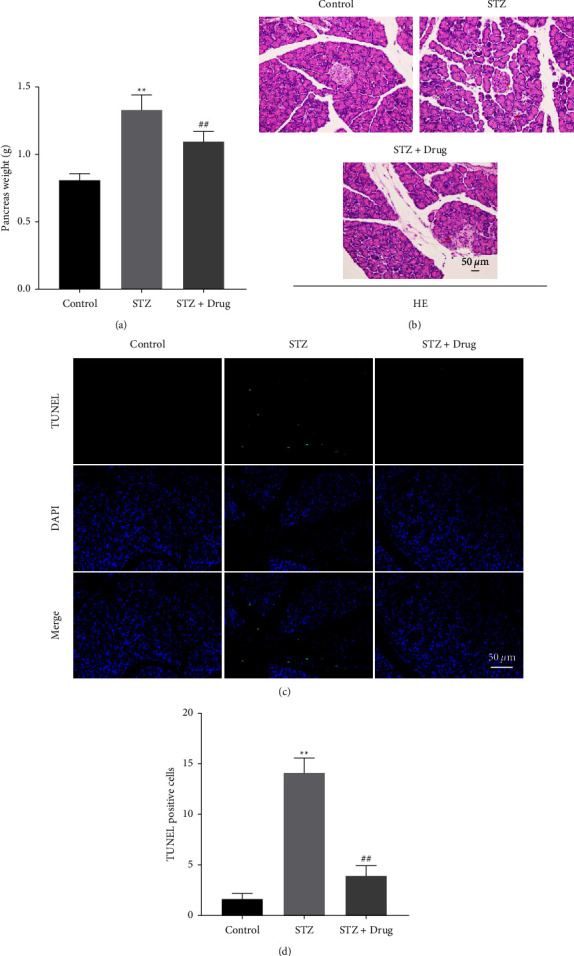
XTZK significantly reversed STZ-induced apoptosis in the pancreatic tissue of rats. (a) The weight of the pancreas in rats was recorded. (b) The injury of the pancreatic tissues in rats was observed by H&E staining. (c), (d) The apoptosis of the pancreatic tissues in rats was observed by TUNEL staining. ^*∗∗*^*P* < 0.01 compared to the control. ##*P* < 0.01 compared to STZ. XTZK, Xiaotangzhike pill; STZ, streptozotocin; H&E, hematoxylin and eosin. Red arrows indicate the inflammatory infiltration in tissues of rats.

To investigate the function of XTZK in Qi and Yin deficiency syndrome of diabetes, *in vivo* model of Qi and Yin deficiency syndrome in diabetes was established. As indicated in Figure 5(a), STZ notably upregulated the weight of the pancreas in rats, while XTZK notably restored the phenomenon. STZ significantly induced the injury of the pancreatic tissues of rats, while the impact of STZ was notably attenuated by XTZK (Figure 5(b)). In addition, STZ notably induced apoptosis in rats, and the apoptotic effect of STZ was greatly inhibited by the XTZK administration (Figures 5(c) and 5(d)). Taken together, XTZK significantly reversed STZ-induced apoptosis in rats.

### 3.6. XTZK Reversed STZ-Induced Apoptosis in Rats through the Mediation of the Akt/GSK-3*β* Signaling

**Figure 6:**
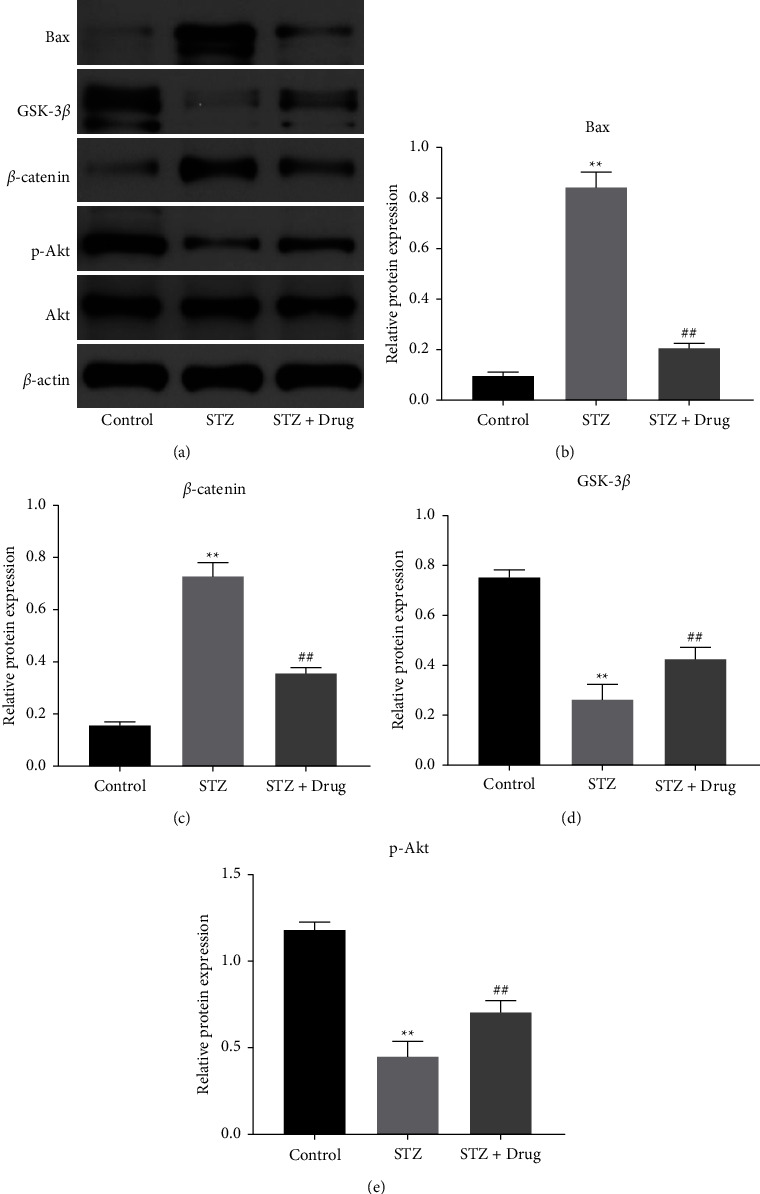
XTZK reversed STZ-induced apoptosis in the pancreatic tissue of rats through the mediation of the Akt/GSK-3*β* signaling. (a) (b), (c) (d), (e) The protein levels of p-Akt, Akt, bax, *β*-catenin, and GSK-3*β* in the pancreatic tissues of rats were investigated by a western blot. The relative expressions were quantified normalizing to Akt or *β*-actin. ^*∗∗*^*P* < 0.01 compared to the control. ##*P* < 0.01 compared to STZ. XTZK, Xiaotangzhike pill; STZ, streptozotocin.

For discovering the mechanism by which XTZK alleviates STZ-induced apoptosis in rats, a western blot was applied. As illustrated in Figures 6(a)–6(c), STZ elevated the levels of Bax and *β*-catenin in tissues of rats, while the impact was alleviated by XTZK. In contrast, the levels of GSK-3*β* and p-Akt in rats were significantly inhibited by STZ, which were rescued by XTZK (Figures 6(a), 6(d) and 6(e)). To sum up, XTZK reversed STZ-induced apoptosis in rats through the mediation of the Akt/GSK-3*β* signaling.

## 4. Discussion

Chinese herbal compounds could inhibit the progression of diabetes. Zhang et al. found that Jiedu Tongluo Tiaogan formula (JDTL) could alleviate the progression of type 2 diabetes mellitus [16]; Yu et al. suggested that Bushen Huoxue prescriptions could inhibit the development of diabetic retinopathy [17]. Meanwhile, XTZK was found to exert an antidiabetic effect [18]. In this research, we found that XTZK could improve the liver function and reverse the injury of the pancreatic tissues in STZ rats. In addition, this study found that XTZK could inactivate p-Smad2 and Akt signaling in STZ rats. Thus, our study first explored the mechanism underlying the function of XTZK in diabetes.

TGF-*β* signaling was verified to be a crucial modulator in diabetes, and it was activated in diabetes [19, 20]. TGF-*β* can activate Smad2 to affect the development of diabetes [21, 22]. This study clarified that XTZK was able to inhibit the phosphorylation of Smad2 in STZ rats. According to the above data, the mechanism underlying the impact of XTZK in diabetes was closely associated with TGF-*β*1 inactivation. According to Jung et al. ethanol extract of *Pharbitis nil* could ameliorate liver fibrosis by the mediation of the TGF-*β*1/Smad2 pathway [23]. TGF-*β* is a vital modulator in liver fibrosis [24, 25]. Furthermore, the progression of diabetes could lead to liver fibrosis [26, 27]. Hence, the similarity between this report and our data might be due to the association between diabetes and renal fibrosis. On the other hand, TGF-*β*1 signaling activation was able to induce the EMT process, and *α*-SMA was considered as the key mediator in the EMT process [28, 29]. Our findings were consistent with these data, showing that XTZK was able to attenuate the severity of diabetes via the inactivation of the TGF-*β*/EMT pathway.

Akt signaling was a crucial modulator in the cell growth, and the phosphorylation of the Akt could lead to cell proliferation [30, 31]. GSK-3*β* was confirmed to be involved in glycogen synthesis, which could decrease blood glucose [32]. In addition, Akt upregulation could increase the level of GSK-3*β* [33]. Thereafter, our work was consistent with these recent reports, verifying that XTZK could inhibit the progression of diabetes through the mediation of the Akt/GSK-3*β* axis.

In general, XTZK was able to attenuate the progression of diabetes *in vivo*. In addition, this study first indicated the function of XTZK in the Akt/GSK-3*β* axis. Therefore, this study was of great significance. Indeed, the mechanism by which XTZK regulates the Akt/GSK-3*β* axis remains unclear. Hence, the detailed mechanism underlying the function of XTZK in the Akt/GSK-3*β* axis will be further investigated in the coming future.

## Data Availability

The data that support the findings of this study are available from the corresponding author upon request.

## Supplementary Materials

Supplementary Materials Supplementary Figure 1: the major components of the XTZK pill. 1: 3′-hydroxypuerarin; 2: puerarin; 3: 3-methoxy puerarin; 4: daidzein.
