# Supplementary material for: Xiaotangzhike Pill Attenuates the Progression of Diabetes In Vivo through the Mediation of the Akt/GSK-3β Axis
Source: Evid Based Complement Alternat Med. 2022 Dec 21;2022:6709506. doi: 10.1155/2022/6709506 (PMC9797293; doi:10.1155/2022/6709506)

**Supplementary Figure 1 The major components of XTZK pill.** 1: 3'-hydroxypuerarin; 2: puerarin; 3: 3-methoxy puerarin; 4: daidzein.


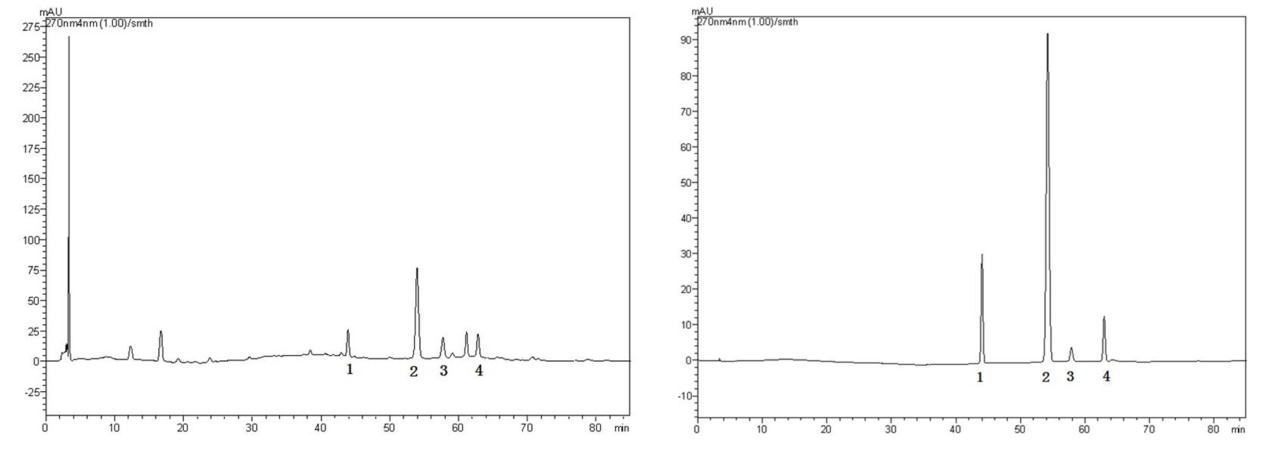

Supplement: Supplementary Materials — Supplementary Figure 1: the major components of the XTZK pill. 1: 3′-hydroxypuerarin; 2: puerarin; 3: 3-methoxy puerarin; 4: daidzein. [file 6709506.f1.docx]
